# Microplastic Shape, Polymer Type, and Concentration Affect Soil Properties and Plant Biomass

**DOI:** 10.3389/fpls.2021.616645

**Published:** 2021-02-16

**Authors:** Yudi M. Lozano, Timon Lehnert, Lydia T. Linck, Anika Lehmann, Matthias C. Rillig

**Affiliations:** ^1^Plant Ecology, Institute of Biology, Freie Universität Berlin, Berlin, Germany; ^2^Berlin-Brandenburg Institute of Advanced Biodiversity Research, Berlin, Germany

**Keywords:** *Daucus carota*, microresp, soil water status, porosity, water-stable aggregates

## Abstract

Microplastics may enter the soil in a wide range of shapes and polymers. However, little is known about the effects that microplastics of different shapes, polymers, and concentration may have on soil properties and plant performance. To address this, we selected 12 microplastics representing different shapes (fibers, films, foams, and fragments) and polymers, and mixed them each with soil at a concentration of 0.1, 0.2, 0.3, and 0.4%. A phytometer (*Daucus carota*) grew in each pot during 4 weeks. Shoot, root mass, soil aggregation, and microbial activity were measured. All shapes increased plant biomass. Shoot mass increased by ∼27% with fibers, ∼60% with films, ∼45% with foams, and by ∼54% with fragments, as fibers hold water in the soil for longer, films decrease soil bulk density, and foams and fragments can increase soil aeration and macroporosity, which overall promote plant performance. By contrast, all shapes decreased soil aggregation by ∼25% as microplastics may introduce fracture points into aggregates and due to potential negative effects on soil biota. The latter may also explain the decrease in microbial activity with, for example, polyethylene films. Our findings show that shape, polymer type, and concentration are key properties when studying microplastic effects on terrestrial systems.

## Introduction

Microplastics (<5 mm) are increasingly reported in terrestrial systems, and due to slow turnover, may be gradually increasing through additions including soil amendments, plastic mulching, irrigation, flooding, atmospheric input, and littering or street runoff (Rillig, 2012; Bläsing and Amelung, 2018; de Souza Machado et al., 2018a; Brahney et al., 2020). Primary microplastics are produced on purpose and used in cosmetic products and various industries, while secondary microplastics are degradation products of larger plastic waste (Wang et al., 2019), which may occur in many shapes, and cover a high physical and chemical diversity (Rillig et al., 2019; Helmberger et al., 2020).

Each microplastic shape may be represented by different polymer types as manufacturers seek to produce plastics with specific properties (e.g., flexibility, roughness, resistance, and durability) (Espí et al., 2006). However, these polymer types are composed of different monomers, which can potentially be hazardous for the environment (Lithner et al., 2011). For instance, polyurethane (PU), a polymer used to produce flexible foams, is made of monomers highly toxic for humans (Lithner et al., 2011) and potentially for soil biota as millions of tons of this plastic are produced annually, potentially increasing its concentration in the soil.

Agricultural soils are particularly prone to being exposed to microplastic, as several pathways for plastic addition and incorporation exist in agroecosystems. For example, fibers are found in soil amended with sewage sludge (Wang et al., 2019). Indeed, microplastic concentrations of 30.7 × 10^3^ particles kg^–1^ dry sludge have been reported (Li et al., 2018). Similarly, plastic mulching is widely used in certain types of agricultural fields (Steinmetz et al., 2016; Bläsing and Amelung, 2018), and thus microplastic film concentrations in soil may increase (Steinmetz et al., 2016). The wide-spread application and the intentional or unintentional ubiquitous distribution of plastics affect even remote landscapes and agricultural sites with plastic-free management plans (Piehl et al., 2018). Other microplastic shapes, such as foams or fragments, can be incorporated into the soil due to littering, street runoff (Bläsing and Amelung, 2018), or wind deposition (Allen et al., 2019; Brahney et al., 2020).

Our knowledge about microplastic effects on terrestrial systems is still scarce and effects reported for plants and soil often seem contradictory, as effects may differ depending on microplastic shape, polymer structure, degradation, additives, and concentration, as well as on the target plant or soil. For instance, microplastic granules of ethylene propylene at 5% may decrease plant biomass likely linked to its polymer composition (van Kleunen et al., 2019), while polyester (PES) fibers at 0.4% may have the opposite effect as microfibers can enhance soil water content and soil aeration (de Souza Machado et al., 2019). PES fibers can also increase soil aggregation as they may help to entangle soil particles (de Souza Machado et al., 2019; Lozano et al., 2020), while opposite effects are detectable for, e.g., polyamide (PA) fibers (de Souza Machado et al., 2019). Likewise, microplastics can affect soil microbial activity as they can increase mortality and histological damage in soil macro-organisms (Huerta Lwanga et al., 2016; Rodriguez-Seijo et al., 2017), and decline richness and diversity of bacterial communities as seen with polyethylene films (Huang et al., 2019; Fei et al., 2020).

As microplastics differ in a number of properties, including shape, polymer type, and concentration, effects on plant species and soils may differ as a function of these properties. To test this, we established a glasshouse experiment that included four microplastic shapes (i.e., fibers, films, foams, and fragments), each of them with three different polymer types and four concentrations (0.1, 0.2, 0.3, and 0.4% w/w). We evaluated effects on shoot and root masses of the plant *Daucus carota*, and on soil aggregation and soil microbial activity. In doing so, we also tested the shape mediation hypotheses proposed by Rillig et al. (2019): at equity of shape effects will be mediated by physical/chemical properties of the particles.

## Materials and Methods

### Species Selection

As microplastics could affect soil water status (de Souza Machado et al., 2019), we selected *D. carota* (wild carrot) as a phytometer. This is a biennial herbaceous typical from dryland ecosystems (Federal Agency for Nature Conservation, 2019) that exhibits clear responses to water availability (Lozano et al., 2019). Seeds of this plant species were obtained from a commercial supplier in the region (Rieger-Hofmann GmbH, Blaufelden, Germany).

### Microplastics

We selected 12 real-world secondary microplastics, representing four microplastic shapes: fibers, films, foams, and fragments, and eight polymer types: PES, PA, polypropylene (PP), low-density polyethylene (LDPE), called polyethylene from now on, polyethylene terephthalate (PET), PU, polystyrene (PS), and polycarbonate (PC) (see additional details in Supplementary Methods S1).

Within each microplastic shape, we selected three microplastics made of different polymers (see details in the Supplementary Material). Fibers, representing those found in agricultural fertilizers such as sewage sludge or compost and films, representing material added to the soil due to temporary greenhouses, plastic mulching, or silage degradation (Piehl et al., 2018; Wang et al., 2019) were manually cut with scissors. A length of 5.0 mm or 5.0 mm^2^, respectively, was established as an upper size threshold in order to generate microplastic fibers and films. Foams (porous, expanded cellular plastic widely used in the packaging industry) and large solid plastics (as, for instance, those related with the degradation of plastic containers) were cut into small pieces by using a Philips HR3655/00 Standmixer (1400 W, ProBlend 6 3D Technologie, Netherlands), sieved through 4 mm mesh, and, if necessary, cut with scissors in order to obtain microplastic foams and fragments (i.e., <5 mm^2^). Microplastics were microwaved (2 min at 500 W) to minimize microbial contamination. Temperature did not approach melting points during microwaving.

### Soil Preparation

We collected dry sandy loam soil (0.07% N, 0.77% C, pH 6.66) from a dry grassland community located in Dedelow, Brandenburg, Germany (53° 37’ N, 13° 77’ W). The soil was sieved (4 mm mesh size), homogenized, and then mixed with each of the microplastics at a concentration of 0.1, 0.2, 0.3, and 0.4% (w/w). Thus, 0.19, 0.38, 0.57, and 0.76 g of each microplastic type were mixed into 190 g of soil for each pot (4 cm diameter, 21 cm height, 200 ml). Soil preparation was done separately for each pot. Microplastics were separated manually and mixed with the soil during 1 min in a large container, before placing it into each individual pot, to help provide an equal distribution of microplastics throughout the soil. Soil was mixed in all experimental units, including the controls, for the same amount of time and with the same intensity, in order to provide the same disturbance.

### Experimental Design

In October 2019, we established the experiment in a glasshouse with a daylight period set at 12 h, 50 klx, and a temperature regime at 22/18°C day/night with a relative humidity of ∼40%. Prior to seedling transplanting, pots were incubated for 2 weeks allowing the interaction between the soil microbial communities and the microplastic particles as well as the potential leaching of plastic components into the soil. During that time, pots were well-watered twice a week by gently spraying 50 ml of distilled water onto the soil surface. Seeds of *D. carota* (∼1000 seeds) were surface-sterilized with 4% sodium hypochlorite for 5 min and 75% ethanol for 2 min and then thoroughly rinsed with sterile water. The seeds were germinated in trays with sterile sand, and individual seedlings of similar size were transplanted into pots 3 days after germination. One seedling was added per pot. Following this, pots were watered for four additional weeks, a sufficiently long period of time to find effects of the treatments on plant and soil properties, as observed in Neal et al. (2012), Rojas-Tapias et al. (2012), or Liang et al. (2019). We thus had 12 microplastic types (i.e., 4 microplastic shapes × 3 polymer types) × 4 concentration levels × 7 replicates = 336 pots. Fourteen additional pots were established as a control without microplastics, which will allow to test the effects of microplastic addition to the soil (added vs not added). All pots were randomly distributed in the greenhouse chamber, and their position shifted twice during the experiment to homogenize environmental conditions. After transplanting, all plants survived until the end of the experiment. At harvest, plants were separated into above and belowground parts; soil was divided into two subsamples of ∼30 g each, one was air-dried and stored at ∼25°C for soil aggregation analyses and the other was kept at 4°C for a maximum of 1 month for soil microbial activity analyses.

### Measurements

#### Biomass

Roots were carefully removed from the soil and gently washed by hand. Then, shoots and roots were dried at 60°C for 72 h, after which their mass was determined.

#### Soil Aggregation

Water-stable soil aggregates (WSA) as a proxy of soil aggregation were measured following a protocol by Kemper and Rosenau (1986), modified as described in Lehmann et al. (2019). Briefly, 4.0 g of dried soil (<4 mm) was placed on small sieves with a mesh size of 250 μm. Soil was rewetted with deionized water by capillarity and inserted into a sieving machine (Agrisearch Equipment, Eijkelkamp, Giesbeek, Netherlands) for 3 min. Agitation and re-wetting cause the treated aggregates to slake. The water-stable fraction (dry matter) was dried and weighed. Subsequently, we extracted the coarse matter which was also dried at 60°C for 24 h. Soil aggregation (i.e., water-stable aggregates) was calculated as: WSA (%) = (dry matter − coarse matter)/(4.0 g − coarse matter).

#### Soil Microbial Activity

We measured soil respiration as it is considered a good proxy of total microbial activity (Gartzia-Bengoetxea et al., 2016). MicroResp^TM^, as described by Campbell et al. (2003), was used to measure community respiration. To do so, we placed approximately 0.42 g of soil into each well of the 96-deep well plates. Four wells were used for each treatment (technical replicates). Soil samples were incubated for 1 day at 25°C prior to carrying out the assay. CO_2_ detection plates were read and then the deep-well plates were sealed with the pre-read CO_2_ detection plates and incubated at 25°C for 6 h in the dark, as recommended by the manufacturer (Macaulay Scientific Consulting, United Kingdom). The change in absorbance values after incubation was then measured on a spectrophotometer microplate reader (Benchmark Plus Microplate Spectrophotometer System, BioRad Laboratories, Hercules, CA, United States) at a wavelength of 570 nm. The CO_2_ rate (μg CO_2_–C g^–1^ h^–1^) per well was calculated using the formula provided in the MicroResp^TM^ manual (Macaulay Scientific Consulting, United Kingdom).

### Statistical Analyses

The effect of microplastic shape, polymer type, and concentration on shoot and root masses, soil aggregation, and microbial activity was analyzed through variance partitioning (using the “vegan” R package), linear models, and multiple comparisons (“multcomp” R package). First, the importance of microplastic shapes, polymer types, and concentration levels in explaining the variation in plant biomass and soil properties was analyzed using variance partitioning “varpart” function. Partition was based on linear regression as the response variables were single vectors (Semchenko et al., 2018). The testable fractions were analyzed with the “anova.cca” function as fractions were expressed as a redundancy analysis model (Oksanen et al., 2019). Then, we performed linear models to test the effect of microplastics on our response variables. Residuals were checked to validate assumptions of normality and homogeneity. When necessary, we implemented the function “varIdent” to account for heterogeneity in the treatment. After this, to the selected model, we implemented the function “glht” and the “Dunnett” test from the “multcomp” R package (Hothorn et al., 2008; Bretz et al., 2011), in order to compare each microplastic treatment with the control (without microplastics). Additionally, effect sizes were estimated to show the variability in the response of our variables, by comparing each microplastic type (i.e., shape and polymer) with the control pots (without microplastics) for each concentration level, using a bootstrap-coupled estimation “dabestr” R package (Ho et al., 2019). Positive effects indicate that the plant trait or soil property values are greater with than without microplastics in the soil. Negative effects indicate the opposite, while neutral effects indicate a similar response with and without microplastics added into the soil.

Finally, we tested the effect of different shapes made of the same polymer base (e.g., PP), on our response variables. As we used real-world microplastics, we could not control the effect of manufacturing additives, which may also play a role. We thus chose the treatments with PP, PET, and polyethylene, as microplastics with different shapes were available for these polymers. We performed an analysis of variance “aov” that included shape and polymer as fixed factor. Shoot mass was log-transformed to meet normality assumptions. Then, we performed multiple comparisons “glht” among treatments by using the “Tukey” test and the function “sandwich” from the eponymous R package; this function provided a heteroscedasticity-consistent estimate of the covariance matrix (Zeileis, 2006; Bretz et al., 2011). Statistical analyses were done in R 3.5.3 (R Core Team, 2019).

## Results

### Shoot Mass

**FIGURE 1:**
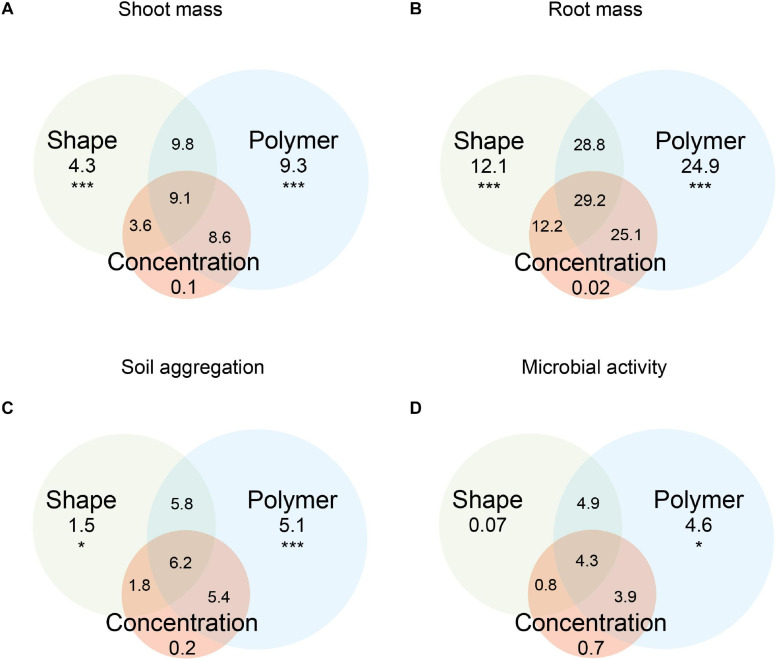
Variation in **(A)** shoot mass, **(B)** root mass, **(C)** soil aggregation, and **(D)** microbial activity explained by microplastic shape, polymer type, concentration, and their combinations. Variance explained is based on adjusted *R*^2^ (*p < 0.05, **p < 0.01, and ***p < 0.001).

**FIGURE 2:**
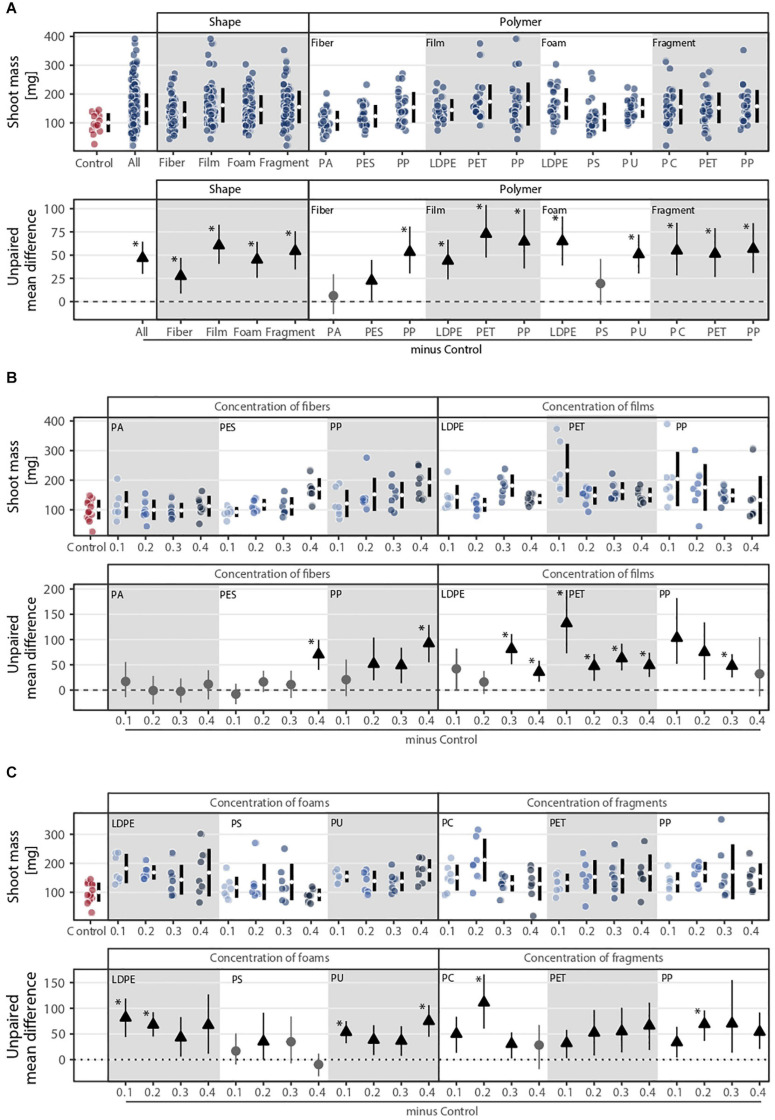
Shoot mass response to **(A)** microplastic shape and polymer type at **(B–C)** different concentrations (0.1, 0.2, 0.3, and 0.4%). That is, **(B)** concentration of fibers and films and **(C)** concentration of foams and fragments. Effect sizes and their variance are displayed as means and 95% confidence intervals. Effects are color-coded: gray circles indicate neutral effect sizes and black arrows with an arrow head pointing upward indicate positive effects; no negative effects were detected. Horizontal dotted line indicates the mean difference value between each microplastic and the control. Polymers: PA (polyamide), PES (polyester), PP (polypropylene), LDPE (low density polyethylene), PET (polyethylene terephthalate), PS (polystyrene), PU (polyurethane), and PC (polycarbonate). Significance was established at 0.05 (Supplementary Tables S1–S3); *n* = 7 for microplastics, *n* = 14 for control samples.

Shoot mass was affected by microplastic shape, polymer type, and concentration (Figures 1A, 2). Shape, polymer type, and their combination were the microplastic properties that explained the most variance in shoot mass (Figure 1A). The effect of microplastic concentration was only relevant when shape and/or polymer type were considered (Figure 1A). Shoot mass increased by ∼27% with fibers, ∼60% with films, ∼45% with foams, and ∼54% with fragments in comparison to the control without microplastics (Figure 2A and Supplementary Table S1). Shoot mass increase was also true for all polymer types except PA and PS whose effects were similar to control (Figure 2A and Supplementary Table S2). PET film was the microplastic that increased shoot mass the most (∼72%), followed by LDPE foams (∼65%) and PP films (∼64%). Fibers increased shoot mass with increasing concentration, a pattern mainly observed with fibers made of PP (Figure 2B and Supplementary Table S2). Microplastic films overall increased shoot mass. However, the trend was contrary compared to fibers (Figure 2B and Supplementary Table S1): the lower the concentration of microplastic films, the more positive the effect shown for PP and PET films (Figure 2B and Supplementary Table S2). Microplastic foams had contrasting effects depending on the polymer type. That is, polyethylene (LDPE) and PS tended to decrease shoot mass with increasing concentration, while PU showed no obvious pattern (Figure 2C and Supplementary Tables S2, S3). Although microplastic fragments overall increased shoot mass, no clear concentration pattern was present (Figure 2C and Supplementary Table S1).

### Root Mass

**FIGURE 3:**
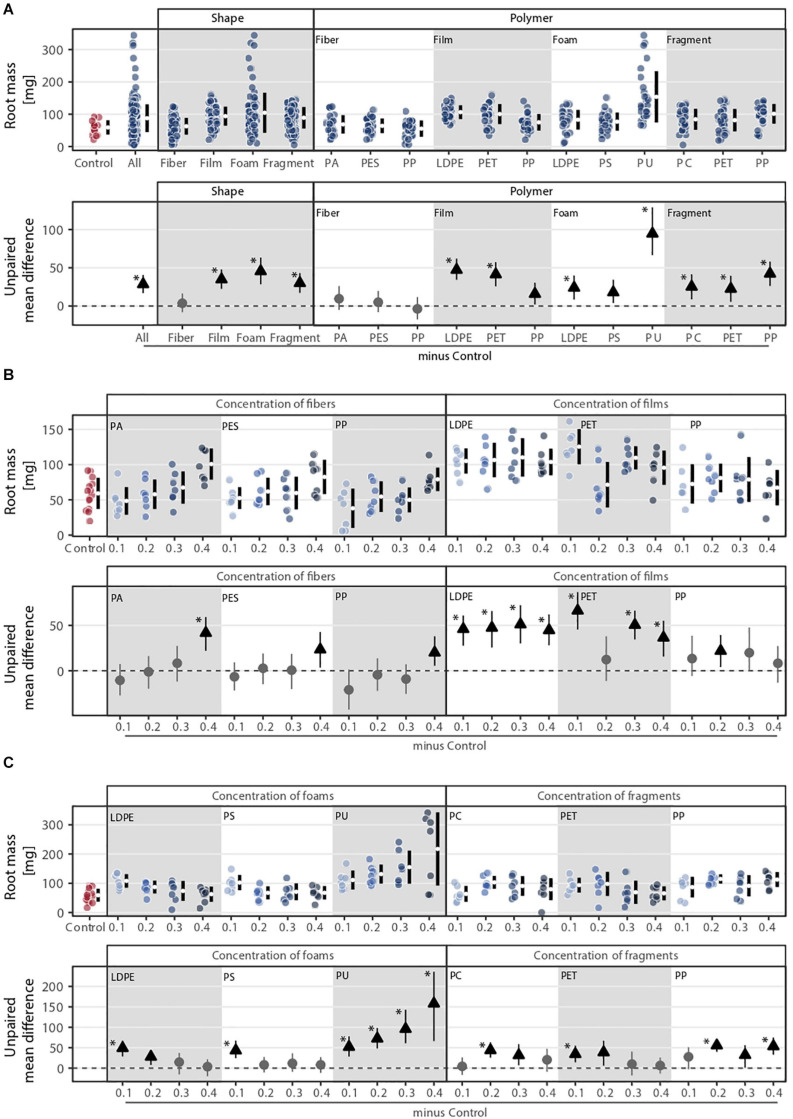
Root mass response to **(A)** microplastic shape and polymer type at **(B–C)** different concentrations (0.1, 0.2, 0.3, and 0.4%). That is, **(B)** concentration of fibers and films and **(C)** concentration of foams and fragments. Effect sizes and their variance aredisplayed as means and 95% confidence intervals. Effects are color-coded: gray circles indicate neutral effect sizes and black arrows with an arrow head pointing upward indicate positive effects; no negative effects were detected. Horizontal dotted line indicates the mean difference value between each microplastic and the control. Polymers: PA (polyamide), PES (polyester), PP (polypropylene), LDPE (low density polyethylene), PET (polyethylene terephthalate), PS (polystyrene), PU (polyurethane), and PC (polycarbonate). Significance was established at 0.05 (Supplementary Tables S1–S3); *n* = 7 for microplastics, *n* = 14 for control samples.

Root mass was affected by microplastic shape, polymer type, and concentration (Figure 3). Shape, polymer type, and their combination were the microplastic properties that explained the most variance in root mass (Figure 1B), which was about double of what these properties explained in terms of shoot mass. The effect of microplastic concentration was also only relevant when shape and/or polymer type were considered (Figure 3A). Foams, films, and fragments increased root biomass by ∼77, 59, and 51%, respectively, while fibers led to a similar biomass to the control (Figure 3A and Supplementary Table S1). PU foam was the microplastic that increased root mass the most (∼160%), followed by LDPE films (∼80%), PP fragments (∼71%), and PET films (∼70%). Although root mass was positively affected by microplastics, the effects diverged from those found for shoot mass. Root mass was only altered by the addition of microfibers under the highest concentration, especially for PA fibers, causing a positive impact (Figure 3B and Supplementary Table S3). Microplastic films and fragments increased root mass irrespective of the concentration (Figures 3B,C), while for foams, we found an increase in root mass as concentration increased for PU; root mass tended to decrease with polyethylene (LDPE) and PS (Figure 3C and Supplementary Table S3).

### Soil Aggregation

**FIGURE 4:**
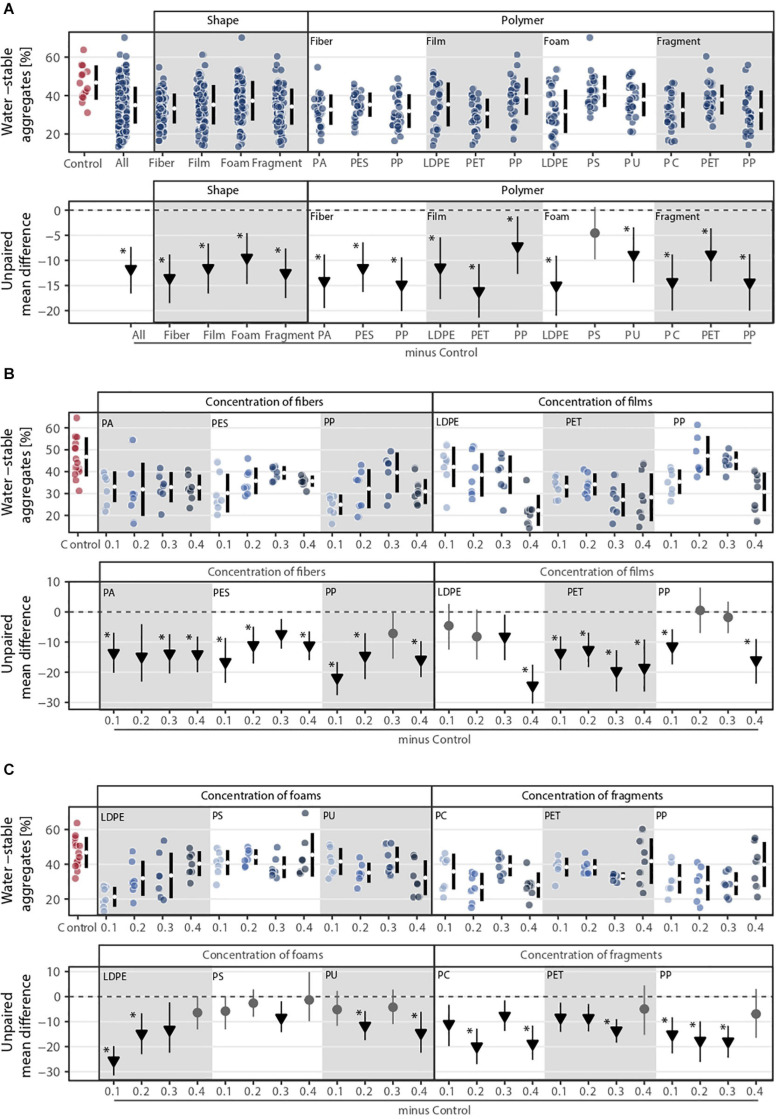
Soil aggregation (i.e., water-stable aggregates) response to **(A)** microplastic shape and polymer type at **(B–C)** different concentrations (0.1, 0.2, 0.3, and 0.4%). That is, **(B)** concentration of fibers and films and **(C)** concentration of foams and fragments. Effect sizes and their variance are displayed as means and 95% confidence intervals. Effects are color-coded: gray circles indicate neutral effect sizes and black arrows with an arrow head pointing downward indicate negative effects; no positive effects were detected. Horizontal dotted line indicates the mean difference value between each microplastic and the control. Polymers: PA (polyamide), PES (polyester), PP (polypropylene), LDPE (low density polyethylene), PET (polyethylene terephthalate), PS (polystyrene), PU (polyurethane), and PC (polycarbonate). Significance was established at 0.05 (Supplementary Tables S1–S3); *n* = 7 for microplastics, *n* = 14 for control samples.

Overall, all microplastic shapes and polymer types negatively influenced soil aggregation (Figure 4). Shape, polymer type, and their combination were the microplastic properties that explained the most variance in soil aggregation (Figure 1C). The effect of microplastic concentration was relevant when polymer type was considered (Figure 1C). Soil aggregation decreased by ∼29% with fibers, ∼25% with films, ∼20% with foams, and ∼27% with fragments in comparison to the control without microplastics (Figure 4A and Supplementary Table S1). Contrary to shoot mass, PET film was the microplastic that decreased the most soil aggregation (∼35%) followed by LDPE foams (∼32%) and PP and PC fragments (∼31%, Figure 4A and Supplementary Table S2). Microplastic fibers consistently reduced the stability of soil aggregates, irrespective of concentration and polymer. For microplastic films and foams, we found concentration dependent trends: microplastic films reduced soil aggregate stability while foams, especially polyethylene (LDPE), had the opposite pattern as concentration increased (Figures 4B,C). Microplastic fragments showed no clear pattern with concentration: PET and PP fragments reduced soil aggregation at lower concentrations but at the highest concentration (0.4%), this effect was neutralized (Figure 4C). PC fragments showed a non-linear concentration effect, with the highest concentration causing strong reduction in soil aggregate stability (Figure 4C). Similar to shoot mass, soil aggregation increased with fibers but decreased with films as concentration increased (Figure 4B).

### Microbial Activity

**FIGURE 5:**
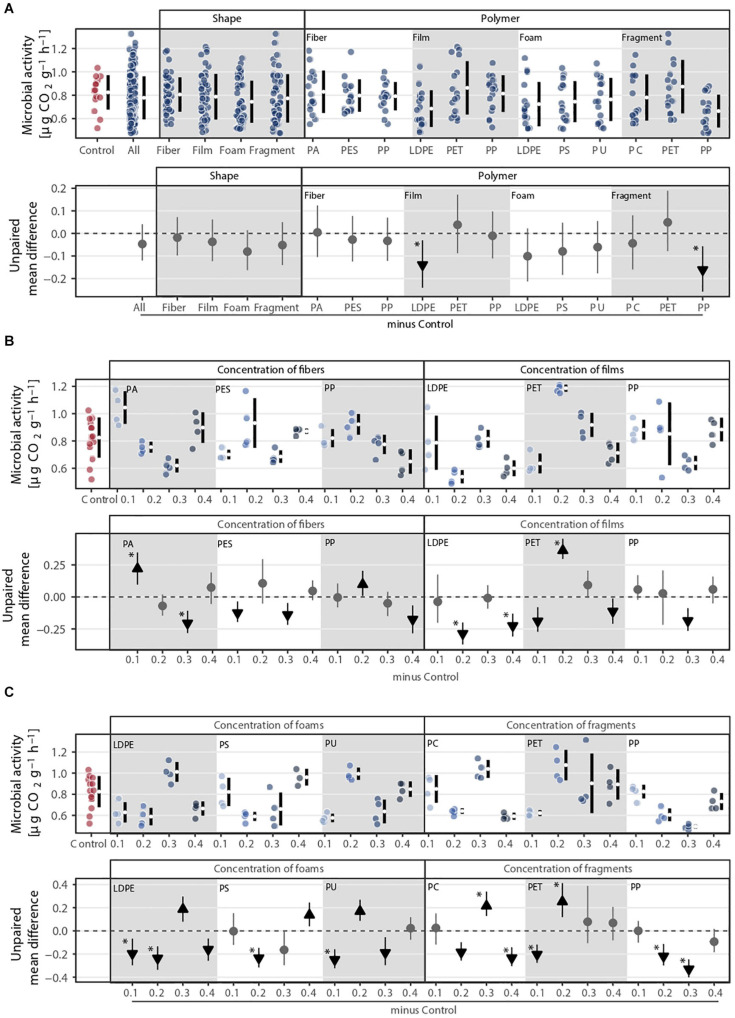
Microbial activity (i.e., respiration) response to **(A)** microplastic shape and polymer type at **(B–C)** different concentrations (0.1, 0.2, 0.3, and 0.4%). That is, **(B)** concentration of fibers and films and **(C)** concentration of foams and fragments. Effect sizes andtheir variance are displayed as means and 95% confidence intervals. Effects are color-coded: gray circles indicate neutral effect sizes and black arrows with an arrow head pointing upward or downward indicate positive or negative effects, respectively. Horizontal dotted line indicates the mean difference value between each microplastic and the control. Polymers: PA (polyamide), PES (polyester), PP (polypropylene), LDPE (low density polyethylene), PET (polyethylene terephthalate), PS (polystyrene), PU (polyurethane), and PC (polycarbonate). Significance was established at 0.05 (Supplementary Tables S1–S3); *n* = 7 for microplastics, *n* = 14 for control samples.

The effect of microplastics on microbial activity was highly variable (Figure 5 and Supplementary Table S1). Only polymer type explained the variance in microbial activity (Figure 1D). PP fragment was the microplastic that decreased microbial activity the most (∼20%), followed by LDPE films (∼17%, Figure 1D and Supplementary Table S2). Regarding concentrations, fibers decreased microbial activity at higher concentrations, i.e., at 0.3% for PA and PES and at 0.4% for PP. By contrast, low concentrations of PA fibers, i.e., 0.1%, increased microbial activity. Microplastic films and foams had an overall neutral or negative effect on microbial activity, respectively (Figures 5B,C). Only PET films at 0.2% concentration and foams made of PU at 0.2%, polyethylene (LDPE) at 0.3%, and PS at 0.4% had a positive effect on microbial activity. Overall, microplastic fragments had a neutral or negative effect, but PC and PET at intermediate values had a positive effect on microbial activity (Figure 5C).

### Microplastic Shape Importance

**FIGURE 6:**
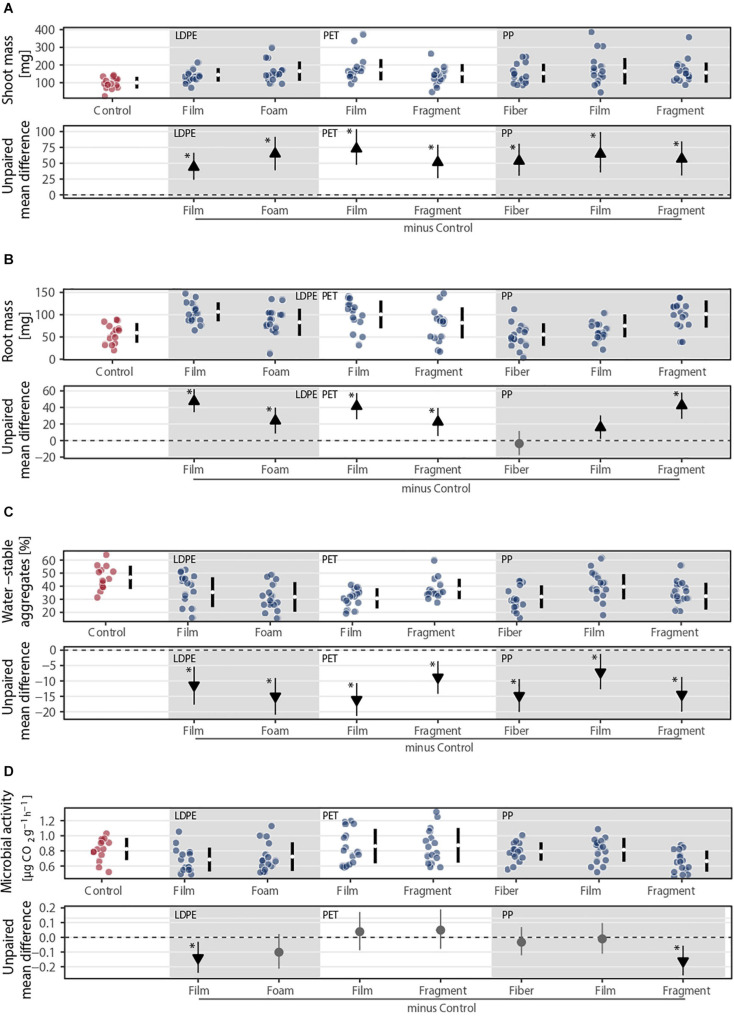
Effects of the same polymer type in different shapes on **(A)** shoot and **(B)** root masses, **(C)** soil aggregation, and **(D)** microbial activity. Effect sizes and their variance are displayed as means and 95% confidence intervals. Effects are color-coded: gray circles indicate neutral effect sizes and black arrows with an arrow head pointing upward or downward indicate positive or negative effects, respectively. Horizontal dotted line indicates the mean difference value between each microplastic and the control. Low density polyethylene (LDPE), polyethylene terephthalate (PET), and polypropylene (PP) were selected as different shapes were made of these polymers. Significance was established at 0.05 (Supplementary Table S4); *n* = 7 for microplastics, *n* = 14 for control samples.

Our results showed the importance of microplastic shape over polymer for different plant traits and soil properties (Figure 6). Although microplastics positively affected shoot mass, we did not find differences among shapes of the same polymer type (Figure 6A and Supplementary Table S4). However, these differences were evident for root mass as polyethylene (LDPE) and PP showed statistically robust differences between shapes. That is, root mass was higher with films compared to foams made of LDPE and gradually increased from fibers to films and to fragments made of PP (Figure 6B and Supplementary Table S4). The key role of shape was also evident in soil aggregation and microbial activity. Soil aggregation was higher with fragments compared to films made of PET and with films compared to fibers made of PP, while microbial activity was lower with fragments compared to films or fibers made of PP (Figures 6C,D and Supplementary Table S4).

## Discussion

Our results clearly showed that microplastic effects on plant traits and soil physical and biological properties depended strongly on polymer type and shape, rather than on concentration. Overall, microplastics in soil increased shoot and root mass by ∼46 and ∼48%, respectively, while decreasing soil aggregation and microbial activity by ∼25 and ∼6%, respectively.

### Microplastics Irrespective of Their Shape Increased Shoot and Root Biomass

Among microplastic shapes, films increased both shoot and root mass by ∼60%, while fibers were the microplastic that increased shoot mass the least (27%); fibers had an almost negligible effect on root mass (6% increase). Shoot mass steadily increased with fiber concentration, becoming noticeable for fibers made of PP and PES, while root mass increased only at highest fiber concentrations. Microfibers decrease soil bulk density (de Souza Machado et al., 2019; Lozano and Rillig, 2020), which cause an increase in soil macroporosity and aeration (Carter and Gregorich, 2006; Ruser et al., 2008). This facilitates root penetration in the soil (Zimmerman and Kardos, 1961) and thus root growth at high microfiber concentration. This increase in root biomass facilitates water and nutrient uptake, an effect that is enhanced by increased water availability, as microfibers enhance water holding capacity (de Souza Machado et al., 2019). Additionally, the increase in root mass might promote rhizodeposition and mycorrhizal associations (Smith and Read, 2010), the latter contributing to the observed increase in shoot mass.

Likewise, the positive effect of microplastic films on shoot and root mass can be linked to the reduction of soil bulk density (Lozano and Rillig, 2020; Yang et al., 2020) and the improvement of associated soil properties (Carter and Gregorich, 2006; Ruser et al., 2008). Shoot and to some extent root mass were affected by microfilm concentration in a pattern opposite to that of microfibers. The decrease in shoot and root mass with microfilm concentration was linked to the creation of more channels for water movement, increasing the rate of soil evaporation (Wan et al., 2019). This water shortage caused a reduction in shoot growth, which in our case was more evident with PP and PET films. Alternatively, the increase in plant growth with microfilms could be linked with the fact that polyethylene (LDPE) films promote Proteobacteria abundance (Huang et al., 2019), a group including plant-growth-promoting phylotypes (Fierer et al., 2007; Hortal et al., 2013). Similarly, shoot and root mass increased with microplastic foams (∼77%) and fragments (∼51%) as potentially soil aeration and macroposity increased, which positively affected plant performance. The sponge-like structured shapes typical of foams can soak up water, potentially increasing water availability for plants.

### Microplastics Irrespective of Their Shape Decreased Soil Aggregation

Microplastics of all shapes, polymer types, and concentration levels decreased soil aggregation by ∼25%. Although fibers were the microplastic that most negatively affected soil aggregation (∼29%), the percentage decrease in this soil property was similar for all microplastic shapes (25.2 ± 1.9%). As microplastics are incorporated into the soil matrix, they prevent microaggregates from effectively being integrated into macroaggregates (Zhang and Liu, 2018), and/or introduce fracture points into aggregates that ultimately decrease aggregate stability. Such negative effects of microfibers on aggregation have been recorded in soil with and without plants (de Souza Machado et al., 2019; Liang et al., 2019). Additionally, as soil biota highly determine soil aggregation (Lehmann et al., 2017), the overall decline in this soil property is also associated with negative effects of microplastics on soil biota. Prior studies have shown that bacterial community diversity declines due to polyethylene (LDPE) films in soil (Fei et al., 2020). Indeed, Actinobacteria, which are one of the bacterial groups that most contribute to soil aggregation (Lehmann et al., 2017), were reduced in abundance and richness due to the presence of microplastic films in soil (Huang et al., 2019; Fei et al., 2020). Even though not addressed here, macro-organisms also contribute to soil aggregation (Bronick and Lal, 2005), and are affected by microplastics in soil. It has been observed that polyethylene and PS particles can be ingested by worms (Huerta Lwanga et al., 2016; Rodriguez-Seijo et al., 2017) and nematodes (Yu et al., 2020), which affect their growth rates and caused histopathological damage, that ultimately affect soil aggregation dynamics.

Importantly, the magnitude of microplastic effects on soil aggregation varied with concentration. There is evidence that soil aggregate stability decreases with microfiber concentration in soil without plants (de Souza Machado et al., 2018b); however, our results show that this does not appear to be the case when introducing a plant species to the test system. We found that soil aggregation tends to increase with microfiber concentration (i.e., PP and to some extent PES), reflecting in part the overall positive effects on root growth, given that roots also contribute to aggregation. Similarly, foams improved soil aggregation with increasing concentration (although always lower than in soil without microplastics), which can be linked with the negative effect that foams have on the number of newly formed aggregates (Lehmann et al., 2020). By contrast, microfilms decreased soil aggregation with increasing concentration, since as discussed above, they can favor water loss from soils, which negatively affected soil aggregation.

### Microplastics of Different Shapes Affects Soil Microbial Activity

Microplastic effects on microbial activity depended on microplastics shape, polymer, and concentration. We expected that microbial activity would be positively correlated with soil aggregation (Bronick and Lal, 2005) and to some extent this was the case as both soil properties decreased with microplastic addition. Polymer type was a key microplastic property explaining the variance in microbial activity. PP fragments (20%) and LDPE films (17%) were the polymers that decreased microbial activity the most. Overall, as soil aggregation decreased with microplastics, the reduction in oxygen diffusion within soil pores and the effects on water flows (Six et al., 2004) explained the reduction in microbial activity as, for instance, with polyethylene (LDPE) films at 0.4% concentration. In accordance with that, Fei et al. (2020) found that microbial activity (measured as FDAse activity) also declined with polyethylene films addition. Likewise, the reduction of microbial activity with microplastic foams at several concentration levels can be related with their chemical properties. Foams (e.g., PU and PS) are made of hazardous monomers (Lithner et al., 2011) that can affect soil biota and thus the soil microbial activity. Indeed, PS foams may contain higher concentrations of organic pollutants (Zhang et al., 2018) not only related to its polymer structure but also related to its shape.

By contrast, we observed that PP films at lower (0.1%) and high (0.4%) concentrations tended to increase microbial activity. A similar pattern was found by Liu et al. (2017) after measuring FDAse activity in soils polluted with PP films. Previous research showed that PP fragments can release dissolved organic carbon and stimulate microbial activity (Romera-Castillo et al., 2018). Similarly, PES fibers at high concentrations tended to increase microbial activity. This aligns with the results of FDA activity (de Souza Machado et al., 2019).

### Shape as a Microplastic Key Property

In this study, we used real-world microplastics which implies different shapes and polymer types but also a variety of additives as different plasticizers, blowing agents, or stabilizers are used to obtain the desired plastic characteristics (flexibility, roughness, density, etc.) (Hahladakis et al., 2018). Our approach allowed us to test the shape mediation hypothesis that states that in addition to the shape, other properties in terms of composition or additives may influence the microplastic effects (Rillig et al., 2019). Here, we show that equal shapes with different properties had a different effect on shoot and root masses and on soil aggregation and microbial activity, as influenced by in this case, the polymer type, additives, and other material properties. Our results showed this for microplastic shapes such as fibers, films, foams, and fragments. In addition, our results lend general support to the shape dissimilarity hypothesis, as our data showed that the more dissimilar microplastics are in shape from the natural population of shapes, the stronger the microplastic effects can be (Rillig et al., 2019). Different shapes of the same polymer type (e.g., fibers, films, and fragments made of PP) affected the response of root mass, soil aggregation, and microbial activity but not of shoot mass. The same was the case for films and fragments made of PET or films and foams made of polyethylene (LDPE). Nonetheless, added to the shape and polymer type, the effects of microplastics additives could also play a role.

As microplastics may come into the soil in different shapes (Rillig et al., 2019), polymer types (Helmberger et al., 2020), and concentrations, it is crucial to understand its effects on soil properties and plant performance, especially as the use of plastic is increasing worldwide. Our findings provide empirical evidence that in the short term (i.e., 4 weeks), microplastics of different shapes and polymers increase shoot and root biomass, but negatively affect soil properties as aggregation and microbial activity. In the long term, additional factors could come into play and negative effects would be more evident. Microplastic effects on plant performance and soil properties will not only depend on the shape, polymer type, and concentration levels, but also on the plant species identity and soil type. For instance, contrary to our results, Qi et al. (2018) found that polyethylene films did not affect biomass of a wheat crop, van Kleunen et al. (2019) found a negative effect of microplastics on plant biomass, while Lozano and Rillig (2020) found that PES fibers may increase biomass of some plant species while decreasing that of others in a grassland community. Likewise, contrary to our results, microfibers may rather promote soil aggregation at the plant community level (Lozano et al., 2020). As plant species can respond differently to microplastic addition, more research is needed in order to understand the effects of shape, polymer type, and concentration levels on plant performance and soil properties in a wide range of plant species and in a variety of soils.

Finally, as microplastics are ubiquitous around the globe, any effects of microplastics on plant-soil systems would have consequences not only in grasslands but also in different ecosystems worldwide. For instance, drylands, one of the largest terrestrial biomes that cover 41% of Earth’s land surface and that supports over 38% of the global human population (Reynolds et al., 2007), characterized by its water scarcity, can be highly threatened with an increasing of microplastic concentration in the soil, especially as microplastics can exacerbate the negative effects that other global change factors as drought have on plant communities (Lozano et al., 2020), soil properties, and ecosystem multifunctionality (Lozano et al., 2021). This in turn may affect ecosystem services (Díaz et al., 2018; Manning et al., 2018) and thus impact various aspects of human well-being. Grasslands, drylands, and other biomes that support many endemic plant species or that are hotspots of biodiversity (MEA, 2005) could experience shifts in plant productivity, diversity, and its associated services with consequences for the population in general. Further research under field conditions has to be performed in order to test these potential effects.

## Data Availability Statement

The original contributions presented in the study are included in the article/Supplementary Material. Further inquiries can be directed to the corresponding author/s.

## Author Contributions

YL and MR conceived the ideas and designed methodology. YL and TL established and maintained the experiment in the greenhouse. LL analyzed the soil aggregation. AL designed the figures. YL analyzed the data and wrote the first draft of this manuscript. All authors contributed to the final version and gave the final approval for publication.

## Conflict of Interest

The authors declare that the research was conducted in the absence of any commercial or financial relationships that could be construed as a potential conflict of interest.
