# Erratum: Microplastic Shape, Polymer Type, and Concentration Affect Soil Properties and Plant Biomass

**DOI:** 10.3389/fpls.2021.714541

**Published:** 2021-06-29

**Authors:** Frontiers Production Office

**Affiliations:** Frontiers Media SA, Lausanne, Switzerland

**Keywords:** *Daucus carota*, microresp, soil water status, porosity, water-stable aggregates

Due to a production error, Supplementary Tables S1–S4 were not published with the original article. The publisher apologizes for this mistake.

In addition, the citation to Supplementary Table S1 should not have appeared in the following sentence from the *Microplastics* sub-section of the *Materials and Methods*:

“We selected 12 real-world secondary microplastics, representing four microplastic shapes: fibers, films, foams, and fragments, and eight polymer types: PES, PA, polypropylene (PP), low-density polyethylene (LDPE), called polyethylene from now on, polyethylene terephthalate (PET), PU, polystyrene (PS), and polycarbonate (PC) (see additional details in Supplementary Methods S1 and Supplementary Table S1).”

The correct sentence should have been as follows “We selected 12 real-world secondary microplastics, representing four microplastic shapes: fibers, films, foams, and fragments, and eight polymer types: PES, PA, polypropylene (PP), low-density polyethylene (LDPE), called polyethylene from now on, polyethylene terephthalate (PET), PU, polystyrene (PS), and polycarbonate (PC) (see additional details in Supplementary Methods S1).”

Furthermore, in the originally published Supplementary Methods, the table showing the “Description and characteristic of the polymer types” was incorrectly labeled as Supplementary Table S1.

The original version of this article and its Supplementary Material have been updated.

