# Supplementary material for: Microplastic Shape, Polymer Type, and Concentration Affect Soil Properties and Plant Biomass
Source: Front Plant Sci. 2021 Feb 16;12:616645. doi: 10.3389/fpls.2021.616645 (PMC7920964; doi:10.3389/fpls.2021.616645)
Supplement: Supplementary file 1 [file Data_Sheet_1.docx]

**Supplementary material**

**Methods S1.**

Fibers: Polyester (PES, Rope Paraloc Mamutec polyester white, item number, 8442172, Hornbach.de), polyamide (PA, connex, item number 10010166, Hornbach.de) and polypropylene (PP, Rope Paraloc Mamutec polypropylene orange, item number, 8442182, Hornbach.de)

Films: Polyethylene low-density (PE, silo film black, folien-bernhardt.de), Polyethylenterephthalat (PET, company: Toppits / product: oven bag), and Cast Polypropylene (PP, company: STYLEX / product: transparent folders).

Foams: Polyurethane (PU, grey foam sheet, item number, 3838930, Hornbach.de), polyethylene (PE, black low density closed cell ETHAFOAM polyethylene foam, Rugen, shrugen.en.alibaba.com), and expandable Polystyrene (PS, EPS70 Insulation Packing Board SLABS, Wellpack Europe).

Fragments: Polyethylenterephthalat (PET, VioStill, item number 41005958, vio.de), Polypropylene (PP, black plastic pots, treppens.de) and Polycarbonate (PC, CD-R Verbatim).

**Description and characteristic of the polymer types. Information extracted from Plastic resins code table (LEED’s) and the polymer properties database (polymerdatabase.com)**

| Polymer | Abbreviation | Description | Product applications |
| --- | --- | --- | --- |
| Polyethylene terephthalate | PET  Mainly known as PES in the fiber industry | PET is clear, tough, and has good gas and moisture barrier properties. This resin is commonly used in beverage bottles and many injection-molded consumer product containers. Cleaned, recycled PET flakes and pellets are in great demand for spinning fiber for carpet yarns, producing fiberfill and geotextiles. Here for the fibers called as Polyester. | Plastic bottles. Food jars for peanut butter, jelly, jam and pickles. Ovenable film and microwavable food trays. In addition to packaging, PET’s major uses are textiles, monofilament, carpet, strapping, films, and engineering moldings. |
| Polyamide | PA | Synthetic polyamides are commonly used in textiles, automotive industry, carpets, kitchen utensils and sportswear due to their high durability and strength. | Applications include almost every industry and market. For example, in the automotive industry, nylons are used for wire and cable jacketing, cooling fans, air intake, turbo air ducts, valve and engine covers, brake and power steering reservoirs, gears for windshield wipers and speedometers. More than 60 percent of the aliphatic polyamides produced are used in commercial fiber applications. This includes carpets, garments, seatbelts, upholstery, ropes and tire reinforcements. |
| Polypropylene | PP | PP has good chemical resistance, is strong, and has a high melting point making it good for hot-fill liquids. This resin is found in flexible and rigid packaging, fibers, and large molded parts for automotive and consumer products. | Containers for food. Bottle caps and closures. In addition to packaging, PP’s major uses are in fibers, appliances and consumer products, including durable applications such as automotive and carpeting. |
| Low-density polyethylene | LDPE | LDPE is used predominately in film applications due to its toughness, flexibility and relative transparency, making it popular for use in applications where heat sealing is necessary. LDPE also is used to manufacture some flexible lids and bottles as well as in wire and cable applications. | Bags for dry cleaning, newspapers, bread, frozen foods, fresh produce, and household garbage. Shrink wrap and stretch film. Coatings for paper milk cartons and hot and cold beverage cups. Container lids. Toys. Squeezable bottles (e.g., honey and mustard). In addition to packaging, LDPE’s major uses are in injection molding applications, adhesives and sealants, and wire and cable coverings. |
| Polyurethane | PU | They can be thermosetting or thermoplastic, rigid and hard or flexible and soft. They are formed from the reaction of an organic diisocyanate with a diol compound, which leads to urethane linkages in the backbone | The versatile urethane including flexible and rigid foams, solid elastomers, extrusion and injection-molded parts, coatings, sealants, and adhesives. Flexible, high-resilience foamed products include mattresses, upholstered furniture, carpet underlays and auto parts like cushions, backs, and armrests. Rigid foamed products with a closed cell morphology are used as insulations for commercial and residential buildings. O Polyurethanes are also used as electrical potting compounds, adhesives, coatings, sealants, and for the fabrication of synthetic fibers (Spandex). |
| Polystyrene | PS | PS is a versatile plastic that can be rigid or foamed. General purpose polystyrene is clear, hard and brittle. It has a relatively low melting point. Typical applications include protective packaging, foodservice packaging, bottles, and food containers. | Food service items, such as cups, bowls, cutlery, hinged takeout containers (clamshells), meat and poultry trays, and rigid food containers (e.g., yogurt). These items may be made with foamed or non-foamed PS. Compact disc cases and aspirin bottles. In addition to packaging, PS’s major uses are in agricultural trays, electronic housings, cable spools, building insulation, video cassette cartridges, coat hangers, and medical products and toys. |
| Polycarbonate | PC | Industry brand known commonly as Lexan. The main advantage of polycarbonate over other types of plastic is unbeatable strength combined with light weight. | A versatile, tough plastic used for a variety of applications, from bulletproof windows to compact disks (CDs). |

**Table S1**. Microplastic shape effect on shoot and root masses, soil aggregation and microbial activity. Results of linear model and multiple comparisons by using the Dunnett test. Values in bold denote a significant effect (p<0.05) of the treatment on the dependent variable. Percentage (%) increase (positive values) or decrease (negative values) of the trait value with respect to the control are shown.

| Linear model | | Shoot mass | |  | Root mass | |  | Soil aggregation | |  | Microbial activity | |  |
| --- | --- | --- | --- | --- | --- | --- | --- | --- | --- | --- | --- | --- | --- |
|  | df | F value | p value |  | F value | p value |  | F value | p value |  | F value | p value |  |
| Treatment | 4 | 10.62 | **<0.001** |  | 21.99 | **<0.001** |  | 8.197 | **<0.001** |  | 0.94 | 0.436 |  |
|  |  |  |  |  |  |  |  |  |  |  |  |  |  |
| Dunnett Test | | Shoot mass | | | Root mass | | | Soil aggregation | | | Microbial activity | | |
| Treatment - control >= 0 | | z value | p-value | % | z value | p-value | % | z value | p-value | % | z value | p-value | % |
| Fibers |  | 2.67 | **0.011** | 27.2 | 0.55 | 0.513 | 6.1 | -5.31 | **<0.001** | -28.8 | -0.41 | 0.97 | -2.2 |
| Films |  | 5.48 | **<0.001** | 60.1 | 5.18 | **<0.001** | 58.9 | -4.36 | **<0.001** | -24.6 | -0.76 | 0.79 | -4.5 |
| Foams |  | 4.32 | **<0.001** | 44.8 | 5.06 | **<0.001** | 76.9 | -3.58 | **<0.001** | -20.2 | -1.69 | 0.22 | -9.7 |
| Fragments | | 5.03 | **<0.001** | 54.0 | 4.32 | **<0.001** | 50.7 | -4.81 | **<0.001** | -26.8 | -1.04 | 0.59 | -6.3 |

**Table S2**. Polymer type effect on shoot and root masses, soil aggregation and microbial activity. Results of linear model, and multiple comparisons by using the Dunnett test. Polypropylene (PP); Polyester (PES); Polyamide (PA); Polyethylene (PE); Polyethylenterephthalat (PET); Polyurethane (PU); Polystyrene (PS); Polycarbonate (PC). Values in bold denote a significant effect (p<0.05) of the treatment on the dependent variable. Percentage (%) increase (positive values) or decrease (negative values) of the trait value with respect to the control are shown.

| Linear model |  |  | Shoot mass | |  | Root mass | |  | Soil aggregation | |  |  | Microbial activity | |  |
| --- | --- | --- | --- | --- | --- | --- | --- | --- | --- | --- | --- | --- | --- | --- | --- |
|  |  | df | F value | p value |  | F value | p value |  | F value | p value |  |  | F value | p value |  |
| Treatment |  | 12 | 6.95 | **<0.001** |  | 12.67 | **<0.001** |  | 6.05 | **<0.001** |  |  | 2.23 | **0.015** |  |
|  |  |  |  |  |  |  |  |  |  |  |  |  |  |  |  |
| Dunnett Test | | | Shoot mass | |  | Root mass | |  | Soil aggregation | |  |  | Microbial activity | |  |
| Treatment - control >= 0 |  |  | z value | p-value | % | z value | p-value | % | z value | p-value | % |  | z value | p-value | % |
| Fibers |  | PP | 3.98 | **<0.001** | 53.1 | -0.48 | 0.98 | -6.2 | -5.00 | **<0.001** | -31.7 |  | -0.66 | 0.75 | -3.9 |
|  |  | PES | 1.94 | 0.10 | 22.3 | 0.68 | 0.72 | 8.4 | -3.87 | **<0.001** | -24.6 |  | -0.52 | 0.80 | -3.3 |
|  |  | PA | 0.57 | 0.78 | 6.3 | 1.19 | 0.47 | 16.1 | -4.74 | **<0.001** | -30.1 |  | 0.07 | 0.95 | 0.5 |
| Films |  | PP | 3.87 | **<0.001** | 64.2 | 2.06 | 0.128 | 26.7 | -2.41 | **0.04** | -15.3 |  | -0.18 | 0.90 | -1.3 |
|  |  | PE | 3.85 | **<0.001** | 43.7 | 6.61 | **<0.001** | 79.9 | -3.82 | **<0.001** | -24.3 |  | -2.50 | **0.05** | -16.9 |
|  |  | PET | 5.05 | **<0.001** | 72.4 | 4.96 | **<0.001** | 70.0 | -5.41 | **<0.001** | -34.3 |  | 0.55 | 0.98 | 4.6 |
| Foams |  | PU | 4.62 | **<0.001** | 50.6 | 5.86 | **<0.001** | 160.3 | -2.99 | **0.01** | -18.9 |  | -1.00 | 0.60 | -7.3 |
|  |  | PE | 4.77 | **<0.001** | 64.6 | 2.89 | **0.01** | 40.4 | -5.04 | **<0.001** | -32.0 |  | -1.63 | 0.29 | -12.2 |
|  |  | PS | 1.49 | 0.347 | 19.2 | 2.25 | 0.08 | 30.2 | -1.53 | 0.24 | -9.7 |  | -1.32 | 0.43 | -9.6 |
| Fragments |  | PP | 4.07 | **<0.001** | 56.3 | 5.12 | **<0.001** | 71.5 | -4.85 | **<0.001** | -30.8 |  | -3.04 | **0.01** | -19.4 |
|  |  | PC | 3.77 | **<0.001** | 54.6 | 2.95 | **0.01** | 42.6 | -4.81 | **<0.001** | -30.5 |  | -0.69 | 0.74 | -5.3 |
|  |  | PET | 3.83 | **<0.001** | 51.1 | 2.54 | **0.04** | 38.0 | -2.97 | **0.01** | -18.9 |  | 0.70 | 0.99 | 5.9 |

**Table S3**. Microplastic concentration effect on shoot and root masses, soil aggregation and microbial activity. Results of linear model, and multiple comparisons by using the Dunnett test. Polypropylene (PP); Polyester (PES); Polyamide (PA); Polyethylene (PE); Polyethylenterephthalat (PET); Polyurethane (PU); Polystyrene (PS); Polycarbonate (PC). Concentration levels: 0.1% to 0.4%. Values in bold denote a significant effect (p<0.05) of the treatment on the dependent variable.

| Linear model |  |  | Shoot mass | | Root mass | | Soil aggregation | | Microbial activity | |
| --- | --- | --- | --- | --- | --- | --- | --- | --- | --- | --- |
|  |  | df | F value | p value | F value | p value | F value | p value | F value | p value |
| Treatment |  | 48 | 4.994 | **<0.001** | 7.283 | **<0.001** | 6.488 | **<0.001** | 9.238 | **<0.001** |
|  |  |  |  |  |  |  |  |  |  |  |
| Dunnett Test | | | Shoot mass | | Root mass | | Soil aggregation | | Microbial activity | |
| Treatment - control >= 0 |  |  | z value | p-value | z value | p-value | z value | p-value | z value | p-value |
| Fibers | PP | 0.1 % | 1.060 | 0.933 | -1.747 | 1.000 | -6.794 | **< 0.001** | -0.067 | 1.000 |
|  |  | 0.2 % | 2.250 | 0.302 | -0.446 | 1.000 | -3.157 | **0.030** | 1.614 | 0.984 |
|  |  | 0.3 % | 2.548 | 0.164 | -1.029 | 1.000 | -1.584 | 0.688 | -0.827 | 1.000 |
|  |  | 0.4 % | 4.498 | **< 0.001** | 2.315 | 0.262 | -4.879 | **< 0.001** | -2.907 | 0.141 |
|  | PES | 0.1 % | -0.732 | 1.000 | -0.799 | 1.000 | -3.893 | **0.002** | -2.055 | 0.773 |
|  |  | 0.2 % | 1.410 | 0.809 | 0.288 | 0.997 | -3.236 | **0.024** | 1.768 | 0.947 |
|  |  | 0.3 % | 0.736 | 0.982 | 0.055 | 0.999 | -2.620 | 0.132 | -2.309 | 0.546 |
|  |  | 0.4 % | 4.358 | **< 0.001** | 2.139 | 0.357 | -4.384 | **< 0.001** | 0.770 | 1.000 |
|  | PA | 0.1 % | 0.876 | 0.967 | -1.123 | 1.000 | -3.457 | **0.011** | 3.654 | **0.012** |
|  |  | 0.2 % | -0.055 | 1.000 | -0.122 | 1.000 | -2.850 | 0.074 | -1.163 | 1.000 |
|  |  | 0.3 % | -0.197 | 1.000 | 0.790 | 0.968 | -3.488 | **0.010** | -3.402 | **0.030** |
|  |  | 0.4 % | 0.733 | 0.982 | 4.071 | **0.001** | -4.087 | **< 0.001** | 1.219 | 1.000 |
| Films | PP | 0.1 % | 2.893 | 0.068 | 1.107 | 0.905 | -3.398 | **0.014** | 0.956 | 1.000 |
|  |  | 0.2 % | 2.422 | 0.216 | 2.262 | 0.289 | 0.146 | 1.000 | 0.456 | 1.000 |
|  |  | 0.3 % | 3.793 | **0.003** | 1.476 | 0.755 | -0.413 | 0.994 | -3.094 | 0.081 |
|  |  | 0.4 % | 1.006 | 0.945 | 0.730 | 0.975 | -3.426 | **0.012** | 0.983 | 1.000 |
|  | PE | 0.1 % | 2.432 | 0.212 | 5.133 | **< 0.001** | -1.004 | 0.929 | -0.625 | 1.000 |
|  |  | 0.2 % | 1.274 | 0.867 | 4.319 | **< 0.001** | -1.882 | 0.504 | -4.758 | **< 0.001** |
|  |  | 0.3 % | 4.848 | **< 0.001** | 4.306 | **< 0.001** | -1.834 | 0.535 | -0.144 | 1.000 |
|  |  | 0.4 % | 3.251 | **0.023** | 4.811 | **< 0.001** | -6.307 | **< 0.001** | -3.739 | **0.009** |
|  | PET | 0.1 % | 3.748 | **0.004** | 5.951 | **< 0.001** | -4.313 | **< 0.001** | -3.144 | 0.070 |
|  |  | 0.2 % | 3.259 | **0.023** | 0.910 | 0.950 | -4.035 | **0.001** | 6.008 | **< 0.001** |
|  |  | 0.3 % | 4.327 | **< 0.001** | 5.885 | **< 0.001** | -4.653 | **< 0.001** | 1.541 | 0.993 |
|  |  | 0.4 % | 3.796 | **0.003** | 3.340 | **0.017** | -3.514 | **0.009** | -1.860 | 0.906 |
| Foams | PU | 0.1 % | 4.637 | **< 0.001** | 3.821 | **0.003** | -1.307 | 0.829 | -4.124 | **0.002** |
|  |  | 0.2 % | 2.387 | 0.232 | 5.309 | **< 0.001** | -3.445 | **0.012** | 2.808 | 0.184 |
|  |  | 0.3 % | 2.357 | 0.247 | 4.329 | **< 0.001** | -1.086 | 0.908 | -3.114 | 0.076 |
|  |  | 0.4 % | 4.388 | **< 0.001** | 3.320 | **0.019** | -3.060 | **0.041** | 0.393 | 1.000 |
|  | PE | 0.1 % | 3.861 | **0.003** | 4.654 | **< 0.001** | -6.707 | **< 0.001** | -3.252 | **0.049** |
|  |  | 0.2 % | 5.274 | **< 0.001** | 2.740 | 0.102 | -3.093 | **0.037** | -3.918 | **0.004** |
|  |  | 0.3 % | 2.011 | 0.445 | 1.044 | 0.922 | -2.444 | 0.196 | 3.110 | 0.077 |
|  |  | 0.4 % | 2.087 | 0.397 | 0.323 | 0.996 | -1.670 | 0.637 | -2.652 | 0.272 |
|  | PS | 0.1 % | 1.034 | 0.939 | 3.752 | **0.004** | -1.479 | 0.747 | -0.043 | 1.000 |
|  |  | 0.2 % | 1.398 | 0.815 | 0.785 | 0.969 | -0.699 | 0.977 | -3.893 | **0.005** |
|  |  | 0.3 % | 1.372 | 0.827 | 0.953 | 0.942 | -2.476 | 0.183 | -2.730 | 0.225 |
|  |  | 0.4 % | -0.781 | 1.000 | 0.793 | 0.968 | -0.379 | 0.995 | 2.021 | 0.800 |
| Fragments | PP | 0.1 % | 2.112 | 0.382 | 1.895 | 0.505 | -3.564 | **0.008** | 0.012 | 1.000 |
|  |  | 0.2 % | 4.297 | **< 0.001** | 6.619 | **< 0.001** | -3.358 | **0.015** | -3.644 | **0.012** |
|  |  | 0.3 % | 1.901 | 0.517 | 2.155 | 0.347 | -4.736 | **< 0.001** | -5.484 | **< 0.001** |
|  |  | 0.4 % | 2.807 | 0.087 | 4.735 | **< 0.001** | -1.362 | 0.805 | -1.548 | 0.992 |
|  | PC | 0.1 % | 2.622 | 0.138 | 0.386 | 0.995 | -2.251 | 0.286 | 0.425 | 1.000 |
|  |  | 0.2 % | 3.774 | **0.003** | 4.303 | **< 0.001** | -4.307 | **< 0.001** | -3.052 | 0.093 |
|  |  | 0.3 % | 2.200 | 0.330 | 2.248 | 0.296 | -2.199 | 0.314 | 3.569 | **0.016** |
|  |  | 0.4 % | 1.212 | 0.889 | 1.333 | 0.823 | -4.661 | **< 0.001** | -3.864 | **0.005** |
|  | PET | 0.1 % | 2.141 | 0.365 | 3.065 | **0.042** | -2.460 | 0.189 | -3.369 | **0.033** |
|  |  | 0.2 % | 2.208 | 0.326 | 2.327 | 0.256 | -2.803 | 0.084 | 4.177 | **0.001** |
|  |  | 0.3 % | 2.258 | 0.297 | 0.624 | 0.984 | -5.700 | **< 0.001** | 1.307 | 1.000 |
|  |  | 0.4 % | 2.569 | 0.156 | 0.606 | 0.985 | -1.035 | 0.921 | 1.142 | 1.000 |

**Table S4.** Effects of the same polymer type from different shapes on shoot and root masses, soil aggregation and microbial activity. Results of analysis of variance (aov), and multiple comparisons by using the Tukey test. Polyethylene (PE), polyethylenterephthalat (PET) and polypropylene (PP) were selected as different shapes were made of these polymers. Values in bold denote a significant effect (p<0.05) of the treatment on the dependent variable.

| Anova (aov) |  | Shoot mass | | Root mass | | Soil aggregation | | Microbial activity | |
| --- | --- | --- | --- | --- | --- | --- | --- | --- | --- |
|  | df | F value | p value | F value | p value | F value | p value | F value | p value |
| Treatment | 6 | 0.802 | 0.57 | 10.97 | **<0.001** | 3.538 | **0.002** | 3.499 | **0.003** |
|  |  |  |  |  |  |  |  |  |  |
| Tukey Test | | Shoot mass | | Root mass | | Soil aggregation | | Microbial activity | |
| Treatment |  | t value | p-value | t value | p-value | t value | p-value | t value | p-value |
| PE | Films vs Foams | 1.437 | 0.777 | -3.384 | **0.014** | -1.208 | 0.888 | 0.643 | 0.994 |
| PET | Films vs Fragments | -1.707 | 0.607 | -2.183 | 0.305 | 3.549 | **0.008** | 0.137 | 1.000 |
| PP | Fibers vs Films | 0.330 | 1.000 | 2.912 | **0.054** | 3.164 | **0.029** | 0.464 | 0.999 |
|  | Fibers vs Fragments | 0.264 | 1.000 | 6.245 | **<0.001** | 0.175 | 1.000 | -2.836 | 0.075 |
|  | Films vs Fragments | -0.107 | 1.000 | 3.571 | **0.007** | -2.758 | 0.088 | -2.935 | **0.058** |

**Raw data.** Shoot and root mass (mg), Microbial activity (CO_2_ (µg CO_2_ g^-1^ h^-1^)), water stable aggregates (WSA (%)); n=7 for all variables except CO_2_ n=4.

| shape | polymer | concentration | shoot | root | CO2 | WSA |
| --- | --- | --- | --- | --- | --- | --- |
| Fibers | PP | 0.1 | 110 | 75 | 0.919464 | 29.06977 |
| Fibers | PP | 0.1 | 73 | 54 |  | 29.36047 |
| Fibers | PP | 0.1 | 189 | 61.2 |  | 26.19048 |
| Fibers | PP | 0.1 | 99 | 46.3 | 0.800037 | 16.76301 |
| Fibers | PP | 0.1 | 86 | 16.6 | 0.773289 | 21.89349 |
| Fibers | PP | 0.1 | 183 | 7 |  | 22.8169 |
| Fibers | PP | 0.1 | 109 | 6 | 0.789853 | 28.31858 |
| Fibers | PP | 0.2 | 130 | 42.4 | 1.010573 | 43.00699 |
| Fibers | PP | 0.2 | 139 | 30.6 |  | 36.32813 |
| Fibers | PP | 0.2 | 276 | 85 |  | 39.86254 |
| Fibers | PP | 0.2 | 136 | 65 | 0.899917 | 25.07645 |
| Fibers | PP | 0.2 | 106 | 33.3 | 0.946827 | 19.28783 |
| Fibers | PP | 0.2 | 135 | 47.2 | 0.830295 | 36.7052 |
| Fibers | PP | 0.2 | 143 | 78.8 |  | 25.21246 |
| Fibers | PP | 0.3 | 169 | 33.7 | 0.671362 | 24.5098 |
| Fibers | PP | 0.3 | 220 | 55 | 0.829732 | 29.42943 |
| Fibers | PP | 0.3 | 146 | 46.7 | 0.809877 | 44.98567 |
| Fibers | PP | 0.3 | 90 | 52 | 0.788577 | 43.98827 |
| Fibers | PP | 0.3 | 181 | 24.6 |  | 40.40698 |
| Fibers | PP | 0.3 | 136 | 58.4 |  | 49.61832 |
| Fibers | PP | 0.3 | 104 | 79.3 |  | 44.34783 |
| Fibers | PP | 0.4 | 254 | 68.5 | 0.718539 | 26.02339 |
| Fibers | PP | 0.4 | 142 | 74.2 |  | 28.74618 |
| Fibers | PP | 0.4 | 193 | 79.13 |  | 24.62006 |
| Fibers | PP | 0.4 | 138 | 69.9 |  | 34.0176 |
| Fibers | PP | 0.4 | 254 | 64 | 0.553802 | 30.9198 |
| Fibers | PP | 0.4 | 210 | 113.6 | 0.733087 | 30.3207 |
| Fibers | PP | 0.4 | 160 | 84.6 | 0.593156 | 41.79104 |
| Fibers | PES | 0.1 | 120 | 46.4 | 0.686493 | 20.91691 |
| Fibers | PES | 0.1 | 102 | 79.7 |  | 39.94253 |
| Fibers | PES | 0.1 | 87 | 61 | 0.753356 | 29.53216 |
| Fibers | PES | 0.1 | 87 | 53.3 | 0.677268 | 25.7971 |
| Fibers | PES | 0.1 | 93 | 51.3 |  | 44.8 |
| Fibers | PES | 0.1 | 95 | 46.9 | 0.686611 | 23.63112 |
| Fibers | PES | 0.1 | 64 | 28.8 |  | 26.83616 |
| Fibers | PES | 0.2 | 107 | 63.5 | 0.780094 | 28.86297 |
| Fibers | PES | 0.2 | 133 | 44.8 | 0.799983 | 33.13783 |
| Fibers | PES | 0.2 | 100 | 46.3 |  | 39.36782 |
| Fibers | PES | 0.2 | 144 | 90.5 | 0.973892 | 39.53488 |
| Fibers | PES | 0.2 | 113 | 50.4 |  | 33.13953 |
| Fibers | PES | 0.2 | 131 | 50 |  | 46 |
| Fibers | PES | 0.2 | 90 | 87.5 | 1.170576 | 30.48433 |
| Fibers | PES | 0.3 | 93 | 81.7 | 0.675771 | 38.0531 |
| Fibers | PES | 0.3 | 97 | 66.8 | 0.661559 | 35.58824 |
| Fibers | PES | 0.3 | 115 | 88 |  | 42.73256 |
| Fibers | PES | 0.3 | 97 | 36.2 | 0.752821 | 41.80791 |
| Fibers | PES | 0.3 | 135 | 67.3 | 0.652379 | 40.40115 |
| Fibers | PES | 0.3 | 75 | 24.2 |  | 35.29412 |
| Fibers | PES | 0.3 | 168 | 53.8 |  | 42.05882 |
| Fibers | PES | 0.4 | 235 | 64.1 | 0.870567 | 36.79525 |
| Fibers | PES | 0.4 | 173 | 95.6 |  | 34.87032 |
| Fibers | PES | 0.4 | 181 | 100.6 |  | 35.31157 |
| Fibers | PES | 0.4 | 174 | 115.8 | 0.843763 | 37.2549 |
| Fibers | PES | 0.4 | 158 | 56.8 |  | 39.13043 |
| Fibers | PES | 0.4 | 115 | 53.1 | 0.883899 | 30.93093 |
| Fibers | PES | 0.4 | 160 | 91.6 | 0.886031 | 35.32934 |
| Fibers | PA | 0.1 | 205 | 52.4 | 0.967238 | 30.96591 |
| Fibers | PA | 0.1 | 97 | 43.7 |  | 22.35294 |
| Fibers | PA | 0.1 | 65 | 34 | 1.106108 | 39.26554 |
| Fibers | PA | 0.1 | 121 | 87.6 |  | 36.31124 |
| Fibers | PA | 0.1 | 144 | 29.1 | 1.18149 | 25.22796 |
| Fibers | PA | 0.1 | 98 | 54.8 | 0.924145 | 39.65015 |
| Fibers | PA | 0.1 | 93 | 37.9 |  | 38.0117 |
| Fibers | PA | 0.2 | 100 | 86.7 |  | 16.85393 |
| Fibers | PA | 0.2 | 155 | 60.6 | 0.716251 | 39.01734 |
| Fibers | PA | 0.2 | 97 | 59.2 |  | 24.55621 |
| Fibers | PA | 0.2 | 82 | 80 | 0.808854 | 55.11364 |
| Fibers | PA | 0.2 | 85 | 42.7 | 0.733561 | 29.71429 |
| Fibers | PA | 0.2 | 49 | 26 | 0.760037 | 29.82456 |
| Fibers | PA | 0.2 | 130 | 50.3 |  | 28.44828 |
| Fibers | PA | 0.3 | 92 | 75.8 |  | 31.15727 |
| Fibers | PA | 0.3 | 146 | 64.6 | 0.603183 | 34.03614 |
| Fibers | PA | 0.3 | 114 | 100.5 | 0.629145 | 42.27405 |
| Fibers | PA | 0.3 | 98 | 53.8 |  | 38.21839 |
| Fibers | PA | 0.3 | 76 | 88.8 | 0.563065 | 34.40233 |
| Fibers | PA | 0.3 | 89 | 56 | 0.68386 | 29.91914 |
| Fibers | PA | 0.3 | 71 | 32.5 |  | 20.4023 |
| Fibers | PA | 0.4 | 125 | 121.6 |  | 41.47059 |
| Fibers | PA | 0.4 | 93 | 84.5 |  | 38.53211 |
| Fibers | PA | 0.4 | 102 | 121 | 0.751833 | 24.03561 |
| Fibers | PA | 0.4 | 166 | 99.4 | 0.950197 | 32.28571 |
| Fibers | PA | 0.4 | 55 | 81.3 |  | 31.73653 |
| Fibers | PA | 0.4 | 108 | 72.1 | 0.877427 | 27.94118 |
| Fibers | PA | 0.4 | 137 | 125.8 | 1.012872 | 33.23171 |
| Films | PE | 0.1 | 229 | 102.7 |  | 38.63636 |
| Films | PE | 0.1 | 147 | 125.8 | 0.800884 | 44.82759 |
| Films | PE | 0.1 | 145 | 76 |  | 42.55952 |
| Films | PE | 0.1 | 140 | 108.8 | 0.589538 | 45.80838 |
| Films | PE | 0.1 | 112 | 115.3 | 1.054496 | 47.26225 |
| Films | PE | 0.1 | 115 | 87.8 | 0.703271 | 52.55255 |
| Films | PE | 0.1 | 117 | 119.8 |  | 23.58209 |
| Films | PE | 0.2 | 147 | 108.8 |  | 35.39823 |
| Films | PE | 0.2 | 133 | 66.5 | 0.506786 | 52.16138 |
| Films | PE | 0.2 | 129 | 90.7 | 0.488019 | 41.90751 |
| Films | PE | 0.2 | 79 | 141.3 | 0.568616 | 30.89888 |
| Films | PE | 0.2 | 101 | 106.5 |  | 30.53892 |
| Films | PE | 0.2 | 101 | 129 | 0.589369 | 50.86705 |
| Films | PE | 0.2 | 126 | 102.7 |  | 28.01205 |
| Films | PE | 0.3 | 214 | 150 | 0.821748 | 33.91813 |
| Films | PE | 0.3 | 149 | 96.2 | 0.905956 | 42.27405 |
| Films | PE | 0.3 | 166 | 84.7 |  | 40.8805 |
| Films | PE | 0.3 | 129 | 77.5 | 0.759563 | 46.72619 |
| Films | PE | 0.3 | 238 | 119.7 |  | 22.91667 |
| Films | PE | 0.3 | 182 | 104.6 | 0.776721 | 48.50299 |
| Films | PE | 0.3 | 193 | 139.1 |  | 34.82143 |
| Films | PE | 0.4 | 123 | 90.4 | 0.566025 | 36.76471 |
| Films | PE | 0.4 | 157 | 87 |  | 22.3133 |
| Films | PE | 0.4 | 138 | 91.7 | 0.679329 | 16.86047 |
| Films | PE | 0.4 | 124 | 97.8 |  | 20.18072 |
| Films | PE | 0.4 | 139 | 115.3 | 0.603841 | 22.70115 |
| Films | PE | 0.4 | 159 | 103.4 | 0.549025 | 22.61905 |
| Films | PE | 0.4 | 114 | 140.9 |  | 14.7541 |
| Films | PET | 0.1 | 180 | 118.8 |  | 26.6289 |
| Films | PET | 0.1 | 336 | 142.6 | 0.74259 | 38.43931 |
| Films | PET | 0.1 | 205 | 119.7 |  | 34.02985 |
| Films | PET | 0.1 | 224 | 137.3 | 0.581512 | 35.88235 |
| Films | PET | 0.1 | 173 | 113.5 | 0.6086 | 32.19814 |
| Films | PET | 0.1 | 133 | 162.5 |  | 26.78063 |
| Films | PET | 0.1 | 378 | 83.9 | 0.608846 | 38.32853 |
| Films | PET | 0.2 | 179 | 34 | 1.214887 | 31.11782 |
| Films | PET | 0.2 | 160 | 68 |  | 29.73761 |
| Films | PET | 0.2 | 149 | 108.3 | 1.201136 | 35.18006 |
| Films | PET | 0.2 | 173 | 123.6 | 1.170449 | 33.88889 |
| Films | PET | 0.2 | 122 | 52.6 |  | 27.94118 |
| Films | PET | 0.2 | 93 | 55.3 | 1.159353 | 40.96045 |
| Films | PET | 0.2 | 159 | 58.6 |  | 40.42553 |
| Films | PET | 0.3 | 147 | 114.3 | 1.006524 | 32.5228 |
| Films | PET | 0.3 | 142 | 101.9 |  | 29.72973 |
| Films | PET | 0.3 | 144 | 95.5 |  | 22.58065 |
| Films | PET | 0.3 | 143 | 91.7 | 0.863991 | 27.76119 |
| Films | PET | 0.3 | 222 | 102.3 |  | 16.52174 |
| Films | PET | 0.3 | 186 | 125.4 | 0.97753 | 21.53846 |
| Films | PET | 0.3 | 158 | 136.6 | 0.821953 | 39.24419 |
| Films | PET | 0.4 | 123 | 101.4 | 0.633857 | 27.32558 |
| Films | PET | 0.4 | 156 | 124.7 |  | 41.51515 |
| Films | PET | 0.4 | 173 | 116.3 | 0.78629 | 22.08955 |
| Films | PET | 0.4 | 144 | 101.2 |  | 43.8806 |
| Films | PET | 0.4 | 190 | 51.5 | 0.76708 | 28.90855 |
| Films | PET | 0.4 | 138 | 94.9 | 0.663519 | 19.11765 |
| Films | PET | 0.4 | 124 | 79.4 |  | 15.08876 |
| Films | PP | 0.1 | 180 | 37.8 | 0.980325 | 41.91617 |
| Films | PP | 0.1 | 172 | 61.6 | 0.886301 | 27.1137 |
| Films | PP | 0.1 | 145 | 70.2 | 0.856776 | 30.40936 |
| Films | PP | 0.1 | 394 | 61.1 | 0.80567 | 38.59649 |
| Films | PP | 0.1 | 214 | 124.3 |  | 41.26506 |
| Films | PP | 0.1 | 214 | 92.4 |  | 35.81662 |
| Films | PP | 0.1 | 109 | 60.3 |  | 32.55814 |
| Films | PP | 0.2 | 136 | 77.8 |  | 40.86957 |
| Films | PP | 0.2 | 218 | 52.7 | 0.536134 | 56.1194 |
| Films | PP | 0.2 | 173 | 96.3 |  | 41.59292 |
| Films | PP | 0.2 | 155 | 112.7 |  | 37.61194 |
| Films | PP | 0.2 | 306 | 81.03 | 0.879098 | 62.00608 |
| Films | PP | 0.2 | 193 | 85.7 | 0.905831 | 50 |
| Films | PP | 0.2 | 49 | 61 | 1.087591 | 42.6087 |
| Films | PP | 0.3 | 134 | 81 | 0.603607 | 51.22699 |
| Films | PP | 0.3 | 154 | 143.2 |  | 42.94118 |
| Films | PP | 0.3 | 146 | 83.7 |  | 46.72365 |
| Films | PP | 0.3 | 164 | 49.9 | 0.671428 | 47.14715 |
| Films | PP | 0.3 | 112 | 61.4 | 0.584179 | 37.57396 |
| Films | PP | 0.3 | 187 | 51 | 0.694323 | 43.62018 |
| Films | PP | 0.3 | 141 | 82.4 |  | 45.50725 |
| Films | PP | 0.4 | 308 | 23.2 | 0.965976 | 33.75394 |
| Films | PP | 0.4 | 142 | 105.2 | 0.776158 | 36.81159 |
| Films | PP | 0.4 | 95 | 77.3 | 0.940079 | 38.43844 |
| Films | PP | 0.4 | 132.67 | 75.8 |  | 40.69767 |
| Films | PP | 0.4 | 86 | 58.5 | 0.853378 | 17.81609 |
| Films | PP | 0.4 | 86 | 75 |  | 23.23529 |
| Films | PP | 0.4 | 79 | 56.2 |  | 24.51253 |
| Foams | PU | 0.1 | 145 | 81.7 | 0.632472 | 34.84419 |
| Foams | PU | 0.1 | 156 | 108.3 |  | 44.86804 |
| Foams | PU | 0.1 | 131 | 77.3 | 0.535826 | 51.63205 |
| Foams | PU | 0.1 | 175 | 121 |  | 35.96491 |
| Foams | PU | 0.1 | 132 | 172.9 | 0.574338 | 41.95402 |
| Foams | PU | 0.1 | 159 | 94.6 | 0.562806 | 50.3012 |
| Foams | PU | 0.1 | 182 | 122.8 |  | 31.6568 |
| Foams | PU | 0.2 | 164 | 94.3 |  | 44.78873 |
| Foams | PU | 0.2 | 110 | 187.4 | 0.963668 | 33.81924 |
| Foams | PU | 0.2 | 108 | 154.1 |  | 26.17647 |
| Foams | PU | 0.2 | 92 | 104.4 | 0.947944 | 39.52096 |
| Foams | PU | 0.2 | 145 | 140.8 |  | 34.50292 |
| Foams | PU | 0.2 | 181 | 130.3 | 0.985439 | 34.67492 |
| Foams | PU | 0.2 | 173 | 109.2 | 1.078169 | 32.64095 |
| Foams | PU | 0.3 | 100 | 148.7 | 0.756125 | 38.7931 |
| Foams | PU | 0.3 | 140 | 246 |  | 33.72093 |
| Foams | PU | 0.3 | 118 | 159.1 |  | 45.64565 |
| Foams | PU | 0.3 | 146 | 108.3 | 0.706021 | 36.68639 |
| Foams | PU | 0.3 | 153 | 97.4 | 0.570012 | 52.01238 |
| Foams | PU | 0.3 | 105 | 214.5 |  | 38.30409 |
| Foams | PU | 0.3 | 198 | 112.2 | 0.516535 | 52.54237 |
| Foams | PU | 0.4 | 184 | 130.6 |  | 34.2029 |
| Foams | PU | 0.4 | 166 | 346.4 | 0.752327 | 45.63953 |
| Foams | PU | 0.4 | 133 | 322.5 | 0.904725 | 21.06825 |
| Foams | PU | 0.4 | 218 | 65.2 | 0.829874 | 29.49853 |
| Foams | PU | 0.4 | 223 | 312.9 |  | 29.09091 |
| Foams | PU | 0.4 | 182 | 66.3 | 0.906438 | 44.91803 |
| Foams | PU | 0.4 | 122 | 278.1 |  | 21.19403 |
| Foams | PE | 0.1 | 224 | 124.8 |  | 27.22063 |
| Foams | PE | 0.1 | 140 | 79.7 |  | 19.21053 |
| Foams | PE | 0.1 | 154 | 133.8 | 0.52973 | 15.42553 |
| Foams | PE | 0.1 | 240 | 105.9 | 0.612518 | 27.64706 |
| Foams | PE | 0.1 | 242 | 135 | 0.612518 | 20.28169 |
| Foams | PE | 0.1 | 148 | 86 |  | 13.41108 |
| Foams | PE | 0.1 | 127 | 92.4 | 0.760671 | 25.94752 |
| Foams | PE | 0.2 | 180 | 103 | 0.528228 | 41.91781 |
| Foams | PE | 0.2 | 155 | 105.8 | 0.690663 | 25.85227 |
| Foams | PE | 0.2 | 216 | 89 |  | 18.13031 |
| Foams | PE | 0.2 | 150 | 44.8 |  | 30.26706 |
| Foams | PE | 0.2 | 155 | 83.2 |  | 31.21547 |
| Foams | PE | 0.2 | 146 | 76.9 | 0.620975 | 48.22888 |
| Foams | PE | 0.2 | 178 | 106.9 | 0.515284 | 28.0236 |
| Foams | PE | 0.3 | 156 | 92.6 | 1.000195 | 20.99125 |
| Foams | PE | 0.3 | 91 | 44.3 |  | 19.49861 |
| Foams | PE | 0.3 | 94 | 15 | 1.019777 | 26.27119 |
| Foams | PE | 0.3 | 236 | 111.2 |  | 54.37352 |
| Foams | PE | 0.3 | 173 | 99.7 | 1.128314 | 33.14286 |
| Foams | PE | 0.3 | 112 | 88.1 |  | 47.42857 |
| Foams | PE | 0.3 | 142 | 66.8 | 0.899547 | 33.23944 |
| Foams | PE | 0.4 | 230 | 38 |  | 40.45584 |
| Foams | PE | 0.4 | 141 | 80.3 | 0.568366 | 46.43875 |
| Foams | PE | 0.4 | 207 | 89 |  | 45.05814 |
| Foams | PE | 0.4 | 303 | 63.6 | 0.676991 | 29.54545 |
| Foams | PE | 0.4 | 70 | 16.8 |  | 35.6546 |
| Foams | PE | 0.4 | 103 | 79.4 | 0.689922 | 35.67416 |
| Foams | PE | 0.4 | 121 | 72.9 | 0.724775 | 49.71264 |
| Foams | PS | 0.1 | 186 | 113.7 | 0.98381 | 43.41317 |
| Foams | PS | 0.1 | 137 | 151.3 |  | 42.44186 |
| Foams | PS | 0.1 | 92 | 108.5 |  | 28.57143 |
| Foams | PS | 0.1 | 76 | 87.2 | 0.879869 | 39.30636 |
| Foams | PS | 0.1 | 119 | 102.6 | 0.730417 | 49.7093 |
| Foams | PS | 0.1 | 115 | 73.8 |  | 35.8209 |
| Foams | PS | 0.1 | 97 | 79.7 | 0.694246 | 47.47774 |
| Foams | PS | 0.2 | 126 | 102.7 |  | 38.73874 |
| Foams | PS | 0.2 | 97 | 76 |  | 39.10448 |
| Foams | PS | 0.2 | 94 | 66.4 | 0.529064 | 50.31056 |
| Foams | PS | 0.2 | 273 | 37.7 | 0.593657 | 43.06785 |
| Foams | PS | 0.2 | 106 | 40.7 | 0.626332 | 42.53968 |
| Foams | PS | 0.2 | 131 | 68.7 |  | 47.24638 |
| Foams | PS | 0.2 | 123 | 78.7 | 0.61191 | 48 |
| Foams | PS | 0.3 | 116 | 65.7 | 0.870789 | 35.90504 |
| Foams | PS | 0.3 | 144 | 58.7 | 0.581826 | 43.19527 |
| Foams | PS | 0.3 | 174 | 122.9 |  | 37.46313 |
| Foams | PS | 0.3 | 71 | 84.1 |  | 35.64955 |
| Foams | PS | 0.3 | 113 | 49.1 | 0.677121 | 50.14925 |
| Foams | PS | 0.3 | 77 | 34 |  | 32.13213 |
| Foams | PS | 0.3 | 253 | 82.4 | 0.511442 | 33.7386 |
| Foams | PS | 0.4 | 92 | 89 |  | 53.06122 |
| Foams | PS | 0.4 | 68 | 60.2 |  | 36.49425 |
| Foams | PS | 0.4 | 90 | 77.6 | 0.886148 | 70.18072 |
| Foams | PS | 0.4 | 93 | 54.7 |  | 34.78261 |
| Foams | PS | 0.4 | 61 | 27.3 |  | 42.67913 |
| Foams | PS | 0.4 | 112 | 95.5 | 0.953406 | 42.97994 |
| Foams | PS | 0.4 | 122 | 68 | 1.044179 | 37.6506 |
| Fragments | PP | 0.1 | 142 | 102.2 | 0.838824 | 31.25 |
| Fragments | PP | 0.1 | 152 | 110.9 |  | 20.34384 |
| Fragments | PP | 0.1 | 81 | 32.8 |  | 26.60819 |
| Fragments | PP | 0.1 | 141 | 94.1 |  | 26.64756 |
| Fragments | PP | 0.1 | 119 | 119.7 | 0.730476 | 41.54303 |
| Fragments | PP | 0.1 | 192 | 107.6 | 0.876923 | 43.9759 |
| Fragments | PP | 0.1 | 112 | 40.2 | 0.855415 | 30.97345 |
| Fragments | PP | 0.2 | 116 | 99.2 | 0.551003 | 41.52542 |
| Fragments | PP | 0.2 | 127 | 101.5 |  | 15.07246 |
| Fragments | PP | 0.2 | 167 | 124.1 | 0.602433 | 33.81924 |
| Fragments | PP | 0.2 | 182 | 123.5 |  | 18.60465 |
| Fragments | PP | 0.2 | 210 | 96 | 0.576663 | 38.94081 |
| Fragments | PP | 0.2 | 195 | 137.7 | 0.690848 | 31.12392 |
| Fragments | PP | 0.2 | 190 | 121.9 |  | 24.86034 |
| Fragments | PP | 0.3 | 137 | 119.2 | 0.484328 | 28.73563 |
| Fragments | PP | 0.3 | 126 | 108.8 |  | 19.77077 |
| Fragments | PP | 0.3 | 357 | 40.3 | 0.47542 | 36.7052 |
| Fragments | PP | 0.3 | 232 | 106.2 |  | 28.96936 |
| Fragments | PP | 0.3 | 157 | 137.9 | 0.499477 | 37.2549 |
| Fragments | PP | 0.3 | 95 | 51.4 |  | 28.48837 |
| Fragments | PP | 0.3 | 91 | 75.6 | 0.518635 | 21.85629 |
| Fragments | PP | 0.4 | 106 | 135.5 |  | 54.13105 |
| Fragments | PP | 0.4 | 162 | 146.3 |  | 27.61628 |
| Fragments | PP | 0.4 | 236 | 118.9 | 0.668476 | 55.89888 |
| Fragments | PP | 0.4 | 131 | 80.6 | 0.742419 | 32.68156 |
| Fragments | PP | 0.4 | 178 | 120.5 | 0.841243 | 42.47788 |
| Fragments | PP | 0.4 | 109 | 108 |  | 44.02332 |
| Fragments | PP | 0.4 | 160 | 79.3 | 0.673893 | 22.02381 |
| Fragments | PET | 0.1 | 111 | 111 |  | 39.76261 |
| Fragments | PET | 0.1 | 86 | 82.5 | 0.644785 | 27.95389 |
| Fragments | PET | 0.1 | 142 | 59.8 |  | 41.59021 |
| Fragments | PET | 0.1 | 173 | 77.1 |  | 38.09524 |
| Fragments | PET | 0.1 | 110 | 138.5 | 0.594786 | 38.36317 |
| Fragments | PET | 0.1 | 139 | 91.4 | 0.647498 | 37.17579 |
| Fragments | PET | 0.1 | 167 | 95.3 | 0.600121 | 45.70637 |
| Fragments | PET | 0.2 | 123 | 93.9 | 1.119385 | 35.01484 |
| Fragments | PET | 0.2 | 236 | 124.2 |  | 39.08046 |
| Fragments | PET | 0.2 | 197 | 72.5 |  | 41.06145 |
| Fragments | PET | 0.2 | 154 | 147.7 | 0.985431 | 35.6546 |
| Fragments | PET | 0.2 | 166 | 118.3 |  | 47.38372 |
| Fragments | PET | 0.2 | 144 | 107.6 | 1.259569 | 34.63687 |
| Fragments | PET | 0.2 | 51 | 21.3 | 0.940413 | 34.80826 |
| Fragments | PET | 0.3 | 187 | 144.2 |  | 29.70588 |
| Fragments | PET | 0.3 | 267 | 86.4 | 0.789482 | 34.16928 |
| Fragments | PET | 0.3 | 79 | 43.2 | 1.32593 | 33.63095 |
| Fragments | PET | 0.3 | 115 | 70 |  | 35.19062 |
| Fragments | PET | 0.3 | 161 | 71.8 |  | 33.94495 |
| Fragments | PET | 0.3 | 150 | 18.8 | 0.743066 | 35.24355 |
| Fragments | PET | 0.3 | 130 | 50.1 | 0.755231 | 30.90379 |
| Fragments | PET | 0.4 | 148 | 54 | 0.871758 | 60.40462 |
| Fragments | PET | 0.4 | 167 | 88.5 | 0.920475 | 47.60479 |
| Fragments | PET | 0.4 | 171 | 44.3 |  | 28.79257 |
| Fragments | PET | 0.4 | 189 | 84.6 | 1.065198 | 36.07038 |
| Fragments | PET | 0.4 | 66 | 36.9 |  | 24.26901 |
| Fragments | PET | 0.4 | 145 | 53.8 | 0.716528 | 41.809 |
| Fragments | PET | 0.4 | 281 | 97.9 |  | 53.71429 |
| Fragments | PC | 0.1 | 149 | 55.9 | 0.816398 | 43.76812 |
| Fragments | PC | 0.1 | 185 | 38.2 |  | 26.09971 |
| Fragments | PC | 0.1 | 219 | 93.5 |  | 27.72861 |
| Fragments | PC | 0.1 | 92 | 37.2 | 0.968249 | 21.63743 |
| Fragments | PC | 0.1 | 144 | 52.1 | 0.679816 | 41.66667 |
| Fragments | PC | 0.1 | 101 | 61 |  | 47.16418 |
| Fragments | PC | 0.1 | 164 | 108 | 0.936732 | 42.72997 |
| Fragments | PC | 0.2 | 198 | 94.7 |  | 32.04748 |
| Fragments | PC | 0.2 | 316 | 131 |  | 24.92754 |
| Fragments | PC | 0.2 | 158 | 101.2 |  | 26.33136 |
| Fragments | PC | 0.2 | 293 | 135 | 0.604135 | 36.28319 |
| Fragments | PC | 0.2 | 102 | 95.4 | 0.639454 | 17.49271 |
| Fragments | PC | 0.2 | 216 | 91.4 | 0.665289 | 35.35032 |
| Fragments | PC | 0.2 | 196 | 73.6 | 0.654876 | 15.90214 |
| Fragments | PC | 0.3 | 135 | 105.4 |  | 31.41994 |
| Fragments | PC | 0.3 | 140 | 132.3 | 1.151469 | 38.8102 |
| Fragments | PC | 0.3 | 162 | 130.7 | 1.059608 | 43.98827 |
| Fragments | PC | 0.3 | 155 | 72.5 |  | 43.65782 |
| Fragments | PC | 0.3 | 77 | 51.6 |  | 34.55657 |
| Fragments | PC | 0.3 | 131 | 90.6 | 0.979514 | 47.38372 |
| Fragments | PC | 0.3 | 117 | 52.5 | 0.967767 | 34.02367 |
| Fragments | PC | 0.4 | 124 | 73.3 |  | 24.40476 |
| Fragments | PC | 0.4 | 134 | 102.1 |  | 29.61877 |
| Fragments | PC | 0.4 | 150 | 108.2 | 0.568761 | 27.91411 |
| Fragments | PC | 0.4 | 194 | 67 | 0.591075 | 17.02128 |
| Fragments | PC | 0.4 | 100 | 85.1 | 0.567887 | 29.39394 |
| Fragments | PC | 0.4 | 180 | 116.8 | 0.640287 | 26.26582 |
| Fragments | PC | 0.4 | 21 | 5.1 |  | 41.36905 |
| Control | Control | 0.4 | 126 | 56.5 | 0.937853 | 36.76471 |
| Control | Control | 0.4 | 139 | 74.5 | 0.67412 | 41.71598 |
| Control | Control | 0.4 | 80 | 39 | 0.764689 | 64.54545 |
| Control | Control | 0.4 | 71 | 34.4 | 0.799568 | 52.81899 |
| Control | Control | 0.4 | 135 | 90.6 | 0.838932 | 40.06024 |
| Control | Control | 0.4 | 128 | 65.5 | 0.528781 | 42.51497 |
| Control | Control | 0.4 | 150 | 70.1 | 1.033912 | 46.51163 |
| Control | Control | 0.4 | 89 | 49.6 | 0.973736 | 55.98802 |
| Control | Control | 0.4 | 31 | 21.8 | 0.908026 | 31.87135 |
| Control | Control | 0.4 | 65 | 52.2 | 0.962224 | 56.45646 |
| Control | Control | 0.4 | 98 | 91.6 | 0.8433 | 51.16279 |
| Control | Control | 0.4 | 94 | 33 | 0.904073 | 39.40299 |
| Control | Control | 0.4 | 110 | 64.8 | 0.589783 | 50.75988 |
| Control | Control | 0.4 | 92 | 84.3 | 0.786727 | 44.04762 |
